# Traumatic Brain Injury Modulates Synuclein-Associated Transcription, Amyloid Plaque Morphology and Cognitive Performance in APPswe/PS1dE9/Blg Mice

**DOI:** 10.3390/biomedicines14071524

**Published:** 2026-07-07

**Authors:** Alina Apostol, Elena Kuzubova, Alexandra Radchenko, Kirill Chaprov, Olesya Shcheblykina, Peter Lebedev, Liliya Korokina, Mikhail Pokrovskii, Valentina Sedinova, Anastasia Khizeva, Natalia N. Ninkina, Mikhail Korokin

**Affiliations:** 1 Institute of Pharmacology of Living Systems, Belgorod State National Research University, Pobedy St., 85, Belgorod 308015, Russia; alinakum835@gmail.com (A.A.); 1015artek1015@mail.ru (E.K.); sandrinkaradchenko@gmail.com (A.R.); shcheblykina@bsuedu.ru (O.S.); lebedev_p@bsuedu.ru (P.L.); korokina@bsuedu.ru (L.K.); pokrovskii@bsuedu.ru (M.P.); 2 Institute of Physiologically Active Compounds, Federal Research Center of Problems of Chemical Physics and Medicinal Chemistry, Russian Academy of Sciences, Chernogolovka 142432, Russia; chapkir@gmail.com (K.C.); sedinova@ipac.ac.ru (V.S.); khizeva@ipac.ac.ru (A.K.); 3 School of Biosciences, Cardiff University, Sir Martin Evans Building, Museum Avenue, Cardiff CF10 3AX, UK

**Keywords:** traumatic brain injury, Alzheimer’s disease, α-, β-, γ-synuclein, neurodegeneration, APP/PS1 transgenic mice, cognitive functions

## Abstract

**Background/Goals:** Traumatic brain injury (TBI) is increasingly recognised as an important risk factor for delayed neurodegeneration and has been implicated in the modulation of Alzheimer’s disease (AD)-related amyloid pathology. However, experimental evidence remains equivocal, suggesting that the effects of TBI on amyloidogenesis are context-dependent and influenced by factors including disease stage, injury severity, and the pre-existing neurodegenerative background. This study aimed to comprehensively assess the effects of TBI on cognitive function, synuclein-family gene expression, neuroinflammatory gene expression and amyloid plaque morphology in APPswe/PS1dE9/Blg mice. **Methods:** Wild-type and APP/PS1 mice were assigned to four experimental groups: WT, WT-TBI, APP/PS1 and APP/PS1-TBI. TBI was induced at 6 months of age using a controlled cortical impact device (precision impactor). Behavioural assessments were conducted at two post-injury time points to evaluate locomotor activity, object recognition memory, short-term spatial memory and spatial learning. Cortex and hippocampus samples were analysed by qRT-PCR to evaluate synuclein-family gene expression and neuroinflammation-related markers. Amyloid plaque pathology was evaluated in Congo red-stained brain sections using QuPath-based image analysis. **Results:** TBI did not induce a consistent increase in amyloid plaque burden in APP/PS1 mice. Instead, TBI was associated with changes in plaque-size distribution, particularly at the later post-injury time point. Behavioural assessments revealed early trauma-associated cognitive impairmen; whereas, impairments observed at later stages appeared to be driven predominantly by progression of the APP/PS1 phenotype. Gene expression analysis revealed region- and genotype-dependent alterations in synuclein-family transcripts and inflammatory markers with the most pronounced changes observed in the cortex. **Conclusions:** These findings indicate that TBI does not uniformly accelerate β-amyloid deposition in APP/PS1 mice with established amyloid pathology. Rather, TBI appears to modify the temporal progression and morphological characteristics of amyloid pathology while interacting with genotype-dependent transcriptional responses involving synuclein-family genes and neuroinflammatory pathways. These results highlight the complex interplay between traumatic injury and pre-existing neurodegenerative processes and warrant further studies at the protein-level and over extended follow-up periods to elucidate the underlying mechanisms.

## 1. Introduction

Traumatic brain injury (TBI) is a leading cause of mortality and disability worldwide, particularly among young and middle-aged individuals, and has been implicated as a significant risk factor for the development of late-life neurodegenerative diseases [1,2]. Every year, tens of millions of people sustain a TBI and the consequences of even mild repetitive injuries may emerge years later as chronic traumatic encephalopathy and cognitive impairment [3,4]. Of particular concern is the fact that moderate and severe TBI increase the risk of dementia by 2.3- and 4.5-fold, respectively, as reported in numerous epidemiological studies [5,6]. Nevertheless, the precise molecular and cellular mechanisms linking acute mechanical brain injury to the delayed progression of amyloid pathology remain incompletely understood.

From a pathophysiological perspective, TBI is a complex biphasic process. Primary injury occurs directly at the moment of impact and includes axonal tearing, contusions and haemorrhages which are largely refractory to therapeutic intervention [7]. Secondary injury develops over minutes, hours and even months following trauma and represents a cascade of metabolic, molecular and cellular events, such as excitotoxicity, oxidative stress, lipid peroxidation, mitochondrial dysfunction and neuroinflammation [8,9]. These delayed processes that open a “therapeutic window” for intervention and, according to current understanding, are closely linked to amyloidogenesis.

Alzheimer’s disease (AD), the most common cause of dementia, is characterised by the accumulation of beta-amyloid (Aβ) peptides in extracellular senile plaques and by hyperphosphorylated tau protein in intracellular neurofibrillary tangles [10,11]. Although familial forms of AD are associated with mutations in the APP, PSEN1 and PSEN2 genes, the vast majority (approximately 90%) of cases are sporadic and their aetiology remains incompletely understood [12,13].

Preclinical studies employing animal models have provided valuable insights into the relationship between TBI and AD. Several major rodent models of TBI have been developed including fluid percussion injury (FPI), controlled cortical impact (CCI) and weight drop models to recapitulate distinct aspects of clinical trauma ranging from focal cortical lesions to diffuse axonal injury [14,15]. When applied to transgenic mouse models of AD (APP/PS1, PDAPP, Tg2576 and APP23/PS45) these approaches have generated important but often conflicting evidence regarding the impact of TBI on amyloid pathology.

On the one hand, several studies demonstrate that TBI can accelerate Aβ accumulation. For instance, repetitive mild traumatic injury in Tg2576 mice has been shown to enhance amyloidogenesis, increase oxidative stress levels, and lead to cognitive impairment [16]. Mechanistically, these effects may be mediated by impaired axonal transport, accumulation of amyloid precursor protein (APP) within damaged axons and enhanced β-secretase (BACE1) activity [17,18]. Furthermore, TBI induces systemic metabolic shifts, including mitochondrial dysfunction and impaired energy metabolism which may further contribute to the progression of AD [19].

Conversely several studies have reported a paradoxical reduction in amyloid plaque burden following TBI. For example, in PDAPP mice with already established amyloidosis injury promoted regression of pre-existing Aβ deposits in the ipsilateral hippocampus, an effect attributed to selective loss of Aβ-producing neurons consistent with the “two-hit” hypothesis [20]. Moreover, in APP/PS1 mice, the impact of TBI on amyloid pathology was found to depend critically on disease stage: injury accelerated amyloidogenesis before plaque onset but reduced plaque burden in animals with established deposits, potentially through enhanced glial-mediated clearance [21].

Thus, the available evidence highlights the complex and multifaceted interplay between TBI and AD pathogenesis. Discrepancies among studies may reflect differences in injury type and severity (e.g., open models involving craniotomy versus closed-head impact models), the transgenic mouse line employed, the age of the animals and corresponding stage of amyloidosis at the time of injury as well as the duration of post-traumatic follow-up [22]. Moreover, the marked heterogeneity of TBI in humans, coupled with the inherent uniformity of experimental models creates a substantial translational gap between promising preclinical findings and the limited success of clinical trials [23].

In light of these considerations, we sought to comprehensively examine the effects of traumatic brain injury in a transgenic mouse model of Alzheimer’s disease. By integrating histological, molecular and behavioural analyses we aimed to define how TBI influences the progression and morphology of amyloid pathology. Elucidating the mechanisms underlying the interplay between TBI and AD may facilitate the development of therapeutic strategies to mitigate cognitive decline following head trauma.

## 2. Materials and Methods

### 2.1. The Laboratory Animals

The study was performed on mice of the APPswe/PS1dE9/Blg (APP/PS1) subline [24,25,26,27,28]. Wild-type C3H/C57BL6 mice were used as controls. Animals were assigned to four experimental groups: WT, WT-TBI, APP/PS1, and APP/PS1-TBI. TBI was induced at 6 months of age. The choice of this age was based on the presence of early pathological features in this mouse line at both morphological and cognitive levels. Each experimental group included 18 animals, consisting of 8 males and 10 females. Animals assigned to the TBI groups were subjected to injury at the same experimental age.

Behavioural testing was performed in two independent post-injury subcohorts. Thirty days after TBI, half of the animals from each group were tested (*n* = 9 per group; 4 males and 5 females, 7 months of age). The remaining animals were tested 60 days after TBI (*n* = 9 per group; 4 males and 5 females, 8 months of age). After completion of the behavioural testing protocol in each subcohort, mice were euthanised, and brains were collected for RNA extraction and histological assessment of amyloid plaque formation using Congo red staining. Thus, molecular and histological analyses were performed in the corresponding 30-day and 60-day post-TBI cohorts. Independent subcohorts were used for each post-injury time point to avoid repeated behavioural testing and repeated tissue sampling. Female mice were tested without consideration of estrous cycle phase [29]. The total number of animals used in the study was 72.

Randomization was carried out prior to the start of the experiment based on body weight and genotype. Data analysis was performed in a blinded manner, with investigators unaware of group allocation until completion of the primary analysis [30,31]. Animals were housed in the specific pathogen-free (SPF) animal house of Belgorod State National Research University under standard conditions, including a standard diet, ad libitum access to food and water, a 12 h light/dark cycle, an ambient temperature of 22–24 °C, and relative humidity of 50–65%. Potential sources of bias in behavioural testing were systematically monitored throughout the study.

#### Modelling of Traumatic Brain Injury

Traumatic brain injury was induced at 6 months of age using a 680999II Precision Impactor system (RWD, China). Mice were anesthetised via intraperitoneal injection of Zoletil 100 (30 mg/kg; Virbac, France) and xylazine (12 mg/kg; Biogel, Belarus). Following scalp incision and skull exposure controlled cortical impact was delivered to the right frontal cortex using a 3 mm impactor tip at 3 m/s. Impact parameters were empirically optimised to prevent skull fracture and haemorrhage. The right frontal cortex was selected as the impact site due to its prominent amyloid burden in this model. The impact coordinates were x = −2.5 and y = +2.

Following injury the incision was closed using sutures and animals were placed in a warmed recovery chamber until full recovery from anaesthesia. Mice were monitored daily for 3 days postoperatively. Exclusion criteria included skull fractures outside the impact site, excessive bleeding, intraoperative death and severe postoperative complications. Post-TBI mortality was 0%.

### 2.2. Genotyping of Transgenic Animals

Genotyping was performed using primers described by Jankowsky et al. [32,33] designed to simultaneously amplify a transgene-specific fragment (when present) and an endogenous mouse genomic DNA fragment serving as an internal control. The reaction included a mixture of three primers: a common reverse primer, PrP_rev (5′-GTGGATACCCCCTCCCCCAGCCTAGACC-3′), homologous to the sequence of the PrP gene in the mouse genome and within the transgene cassette; a forward primer, PrP_for (5′-CCT CTT TGT GACTATGTGGACTGATGTCGG-3′), homologous to a region of the genomic mouse PrP that was removed from the MoPrP. Xho vector; and specific forward primers for PS1 (5′-CAG GTG GTG GAG CAA GAT G-3′) and APP (5′-CCG AGA TCT CTGAAGTGAAGATGGATG-3′), homologous to the sequences of the mutant proteins within the transgene cassette. The amplification protocol was as follows: 1 cycle—95 °C for 3 min; 30 cycles—95 °C for 20 s, 55 °C (for APP) or 65 °C (for PS1) for 20 s, 72 °C for 20 s; and 1 cycle—72 °C for 2 min.

### 2.3. Behavioural Testing

To avoid potential learning effects associated with the short interval between experimental time points, animals were divided into two independent cohorts. No mouse underwent repeated behavioural testing.

#### 2.3.1. Open Field Test

Animals were assessed in an Open Field apparatus (OpenScience, Krasnogorsk, Russia) and their spontaneous locomotor activity was recorded. The apparatus consisted of a 50 × 50 cm square chamber made of opaque plexiglass. Locomotor behaviour was quantified using automated software which measured distance travelled, activity and mean velocity (cm/s). Each animal was tested for 5 min under standard room lighting conditions [34,35,36,37].

#### 2.3.2. Novel Object Recognition Test

This behavioural paradigm is based on the innate exploratory behaviour of rodents and consists of three phases: habituation, training/adaptation and testing. On the first day animals were placed in an empty 50 × 50 cm arena (OpenScience, Krasnogorsk, Russia) and allowed to freely explore for 5 min under standard room lighting conditions. On the second day (adaptation phase) animals were reintroduced to the same arena containing two identical objects. On the third day (testing phase) animals were exposed to one familiar object from the previous session and one novel object. The following parameters were recorded: the number of approaches to the novel and familiar objects and the time spent exploring each object [24,33,34,36].

#### 2.3.3. Y-Maze

Working memory was assessed using a Y-maze (OpenScience, Krasnogorsk, Russia) with arm dimensions of 32.5 × 8.5 × 15 cm (L × W × H). The test was conducted under standard room lighting (40 lux). Mice were first allowed to explore two arms of the maze for 5 min, while the third arm was blocked. After a 30 min inter-trial interval, a second trial was conducted, during which the mice were allowed to explore all three arms for 5 min total. An entry into an arm was recorded when more than half of the mouse’s body crossed the boundary between two arms. The number of entries and the time spent in each arm were recorded. Analysis was performed using two scenarios: the entire 5 min test duration, or the first 2 min of “active exploration” [24,33,34].

#### 2.3.4. Barnes Maze Test

This test was used to assess spatial learning and memory in rodents. The Barnes Maze (OpenScience, Krasnogorsk, Russia) evaluates the ability of mice to explore the environment and locate an escape hole using distal visual cues placed around the testing arena. The apparatus consists of a circular platform (122 cm in diameter) containing 40 evenly spaced holes (5 cm in diameter), one of which leads to an escape box. Distal visual cues consisted of four black-and-white geometric patterns positioned at the north, south, east, and west locations. Behaviour was recorded for 5 min using a video tracking system. Outcome measures included total distance travelled, mean movement velocity, and latency to locate the escape hole within the allotted time [38,39].

### 2.4. Analysis of Gene Expression

Cortical and hippocampal samples were snap-frozen on dry ice and stored at −80 °C until further processing as previously described. Total RNA was isolated from brain tissue using Extract RNA reagent (Eurogen, Moscow, Russia). Tissue homogenates were prepared and mixed with chloroform (Scharlab, Barcelona, Spain) followed by centrifugation at 12,000× *g* for 20 min (Eppendorf, Hamburg, Germany). The upper aqueous phase was collected and mixed with an equal volume of cold isopropanol (Panreac Applichem, Barcelona, Spain). After incubation on ice for 30 min samples were centrifuged at 12,000× *g* for 15 min. The resulting RNA pellets were washed twice with 76% ethanol, air-dried and resuspended in RNase-free water [22].

The RNA concentration was measured using a NanoDrop 2000c spectrophotometer (NanoDrop Technologies, Wilmington, DE, USA). Reverse transcription was performed using the First Strand cDNA Synthesis MMLV Kit (Evrogen, Moscow, Russia) according to the manufacturer’s instructions.

Real-time PCR (qRT-PCR) was performed using HS-qPCR SYBR Blue (Biolabmix, Moscow, Russia). Each sample was analysed using a 384-well qRT-PCR detection system DTprime5 (“DNA-Technology”, Moscow, Russia). The amplification reaction was performed in a final volume of 10 μL. The cycling protocol consisted of an initial denaturation step at 95 °C for 5 min, followed by 38 cycles of denaturation at 95 °C for 20 s, annealing at 60 °C for 20 s, and extension at 72 °C for 30 s. This was followed by a melting curve analysis consisting of denaturation at 95 °C for 5 s and 60 cycles at 65 °C for 15 s. Primers for *Gapdh*, *Snca*, *Sncb*, *Sncg*, *Gfap*, and *Il1b* were purchased from Eurogen (Moscow, Russia). The specificity of primers targeting synuclein-family transcripts was validated using DNA templates derived from neuronal tissues of mice with different combinations of synuclein-family gene knockouts [27]. Primers for *Tnf* and *Gsk3b* were purchased from Sigma-Aldrich (Burlington, MA, USA). All samples were analysed in triplicate, and the data were normalised to *Gapdh* mRNA expression. Target genes and primer sequences are listed in Table 1.

### 2.5. Histological Studies

**Figure 1:**
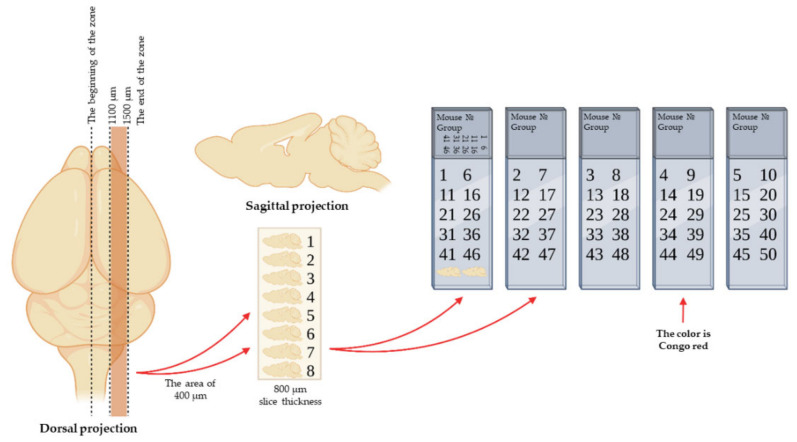
Schematic representation of brain sectioning for histological preparation. Five slides, each containing 10 brain sections, were prepared from a 400 µm brain region. Every fifth serial section was allocated to a separate slide. Modified from Kuzubova et al. [44].

Animals were euthanised by overdose and brains were rapidly extracted and fixed in Carnoy’s solution (96% ethanol, chloroform, and glacial acetic acid in a 6:3:1 ratio) [40,41]. Serial histological sections (7–8 µm) were then prepared according to a standard protocol and stained with Congo red [42,43,44]. The experimental scheme outlining the organisation of section groups for morphometric analysis of inclusions is shown in Figure 1.

Microscopy of the specimens was performed using a Nikon Eclipse Ti (Amstelveen, The Netherlands) microscope equipped with a motorised stage. Panoramic imaging of the mouse brain sections in TRITC fluorescence mode was conducted with a 10× objective using the NIS Elements AR software (version 4.6, Laboratory Imaging, Prague, Czech Republic), with frame stitching into a single composite image, 10% overlap, and automatic post-processing.

The resulting images were uploaded into QuPath software (version 0.5.1, Belfast, Northern Ireland, UK) for the detection and analysis of aggregates. Object detection was based on a fluorescence intensity threshold applied to amyloid-positive spots relative to background signal in intact brain tissue (threshold range: 90–100; smoothing sigma: 1). Incorrect detections were manually corrected.

Plaque size was automatically quantified by QuPath and expressed in µm^2^. Plaque density was calculated as the number of plaques per analysed tissue area and expressed as plaques/mm^2^. Plaques were then assigned to predefined size categories according to their area: <100 µm^2^, 100–200 µm^2^, 200–500 µm^2^, and >500 µm^2^.

To confirm the reproducibility of the results calculations were carried out by two independent researchers [34,45]. The two counts differed by less than 5%, indicating high consistency between measurements.

### 2.6. Statistical Analysis

Statistical analyses were performed using GraphPad Prism 8.0 (GraphPad Software, San Diego, CA, USA). Data are presented as mean ± SEM unless otherwise stated. Normality of distribution was assessed using the Shapiro–Wilk test. For normally distributed data group comparisons were performed using two-way ANOVA with genotype and TBI as main factors, followed by Tukey’s post hoc multiple-comparisons test when appropriate. For non-normally distributed data, the Kruskal–Wallis test followed by Dunn’s post hoc multiple-comparisons test was applied.

For behavioural tests involving repeated measurements across training days such as the Barnes maze acquisition phase repeated-measures two-way ANOVA or a mixed-effects model was used with day as the within-subject factor and experimental group as the between-subject factor. If missing values were present a mixed-effects model was applied. Post hoc comparisons were corrected for multiple testing.

Correlation analyses between behavioural parameters and amyloid pathology were performed using Pearson’s correlation for normally distributed data and Spearman’s rank correlation for non-normally distributed data. Statistical significance was set at *p* < 0.05. Results with 0.05 ≤ *p* < 0.10 were considered statistical trends rather than significant effects. No data points were excluded unless they met predefined technical exclusion criteria.

## 3. Results

### 3.1. Evaluation of Gene Expression

The molecular mechanisms linking acute traumatic injury to chronic neurodegeneration remain poorly understood. Of particular interest is the synuclein family (*Snca*, *Sncb*, *Sncg*), which is implicated in synaptic function and neuroinflammatory processes; however, its coordinated response to TBI in the context of amyloid pathology has not been investigated. In this study we examined the effects of TBI on the expression of *Snca*, *Sncb*, *Sncg,* the astrocytic marker *Gfap*, the pro-inflammatory cytokines *Il1b* and *Tnf* and glycogen synthase kinase-3β (*Gsk3b*) in the cortex and hippocampus at two post-injury time points: 30 and 60 days after TBI.

**Figure 2:**
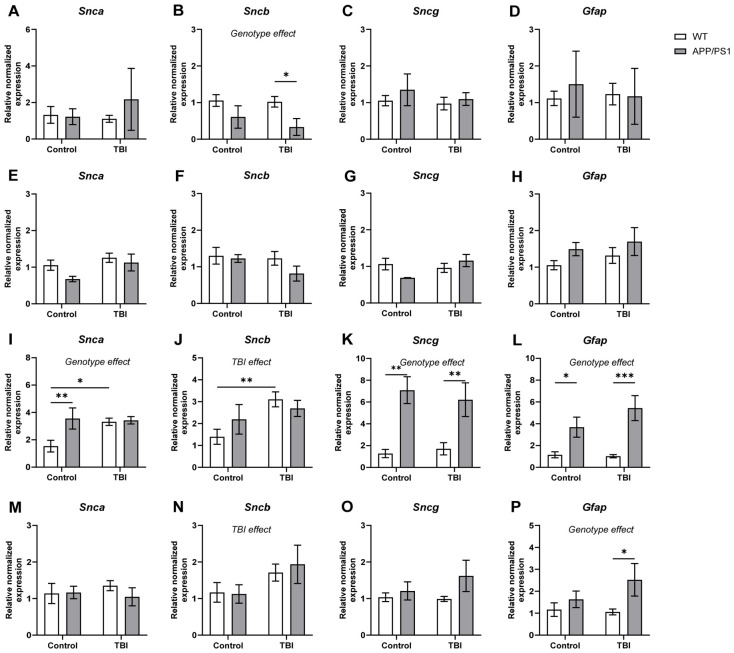
Relative normalised expression of *Snca*, *Sncb*, *Sncg*, and *Gfap* in cortex and hippocampus of WT and APP/PS1 mice 30 and 60 days after TBI. WT, white bars; APP/PS1, grey bars. (**A**–**D**) Cortex, 30 days after TBI; (**E**–**H**) hippocampus, 30 days after TBI; (**I**–**L**) cortex, 60 days after TBI; (**M**–**P**) hippocampus, 60 days after TBI. * *p* < 0.05, ** *p* < 0.01, *** *p* < 0.001 (two-way ANOVA).

In the cortex, 30 days after TBI, a significant effect of genotype on *Sncb* expression was detected (F = 7.745, *p* = 0.0123; two-way ANOVA; Figure 2B). APPswe/PS1dE9/Blg (APP/PS1) mice subjected to TBI exhibited reduced *Sncb* expression compared with TBI-exposed wild-type animals. No significant differences between groups were observed for *Snca*, *Sncg* or *Gfap* expression in the cortex at this age (Figure 2A,C,D). Likewise, no significant group differences were detected in the hippocampus 30 days after TBI for any of the analysed genes (Figure 2E–H).

In the cortex, 60 days after TBI, two-way ANOVA revealed a significant effect of genotype on *Snca* expression (F = 4.956, *p* = 0.0342; Figure 2I). APP/PS1 mice without TBI exhibited higher *Snca* expression than WT controls. Following TBI, *Snca* expression in APP/PS1-TBI mice showed a trend toward convergence with WT-TBI values, although this effect did not reach statistical significance (F = 2.978, *p* = 0.0954; Figure 2I). For *Sncb* expression a significant effect of TBI was observed in WT animals (F = 6.636, *p* = 0.0152; Figure 2J) with higher expression in WT-TBI mice than in WT controls. *Sncg* expression showed a strong effect of genotype (F = 22.87, *p* < 0.0001; Figure 2K) with approximately 50% higher levels in APP/PS1 mice compared with WT controls. In transgenic mice both APP/PS1 control and APP/PS1 TBI groups exhibited increased *Sncg* expression compared with WT control and WT TBI groups. Similarly, *Gfap* expression in the cortex 60 days after TBI showed a significant effect of genotype (F = 20.57, *p* < 0.0001; two-way ANOVA; Figure 2L) with both APP/PS1 control and APP/PS1 TBI mice displaying higher expression levels than their corresponding WT groups. As in the cortex a significant genotype effect on *Gfap* expression was observed in the hippocampus 60 days after TBI (F = 5.998, *p* = 0.0214; Figure 2P) where APP/PS1 TBI mice showed increased *Gfap* expression compared with WT TBI mice. In contrast, no significant differences were detected for *Snca*, *Sncb* or *Sncg* expression in the hippocampus at this time point (Figure 2M–O).

Our findings on synuclein expression add an additional layer of complexity to the interaction between TBI, amyloidogenesis and neurodegeneration. A significant genotype-dependent effect on *Snca* expression was observed in the cortex 60 days after TBI: in APP/PS1 mice without TBI, *Snca* levels were elevated compared with WT controls; whereas, following injury, *Snca* expression in APP/PS1 mice became comparable to that of WT mice while remaining higher than in WT controls. This convergence may reflect either an exhaustion of compensatory responses or injury-induced dysregulation of α-synuclein expression in the context of pre-existing Aβ pathology. In addition, in WT animals TBI was associated with increased cortical *Sncb* expression; whereas, this effect was absent in APP/PS1 mice, suggesting genotype-dependent differences in molecular response to injury. The most pronounced changes were observed for *Sncg*. In the cortex of APP/PS1 mice *Sncg* expression was approximately 50% higher than in WT animals, both under baseline conditions and following TBI. Accordingly, in the transgenic line both injury and its absence were associated with persistently elevated *Sncg* levels relative to WT groups. The lack of significant changes in *Snca*, *Sncb* and *Sncg* expression in the hippocampus at this time point suggests a region-specific synuclein response in the context of combined amyloid pathology and trauma. Given that α- and γ-synucleins are involved in the regulation of synaptic transmission, vesicular trafficking, and protein aggregation the observed expression changes may reflect an adaptive attempt to maintain synaptic homeostasis under the combined influence of Aβ pathology and TBI, or alternatively may contribute to the development of a phenotype with increased vulnerability to neurotoxic stress [5,21].

**Figure 3:**
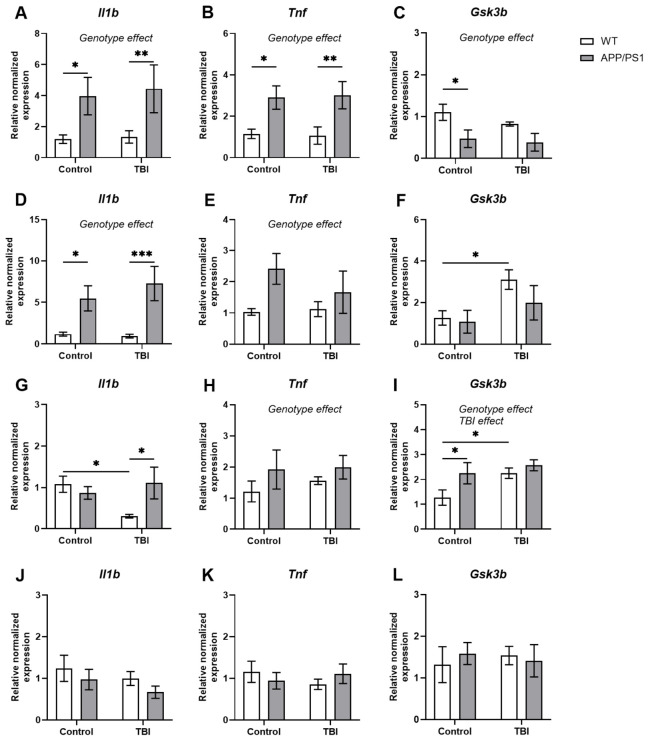
Relative normalised expression of neuroinflammation-related genes (Il1b, *Tnf* and *Gsk3b*) in the cortex and hippocampus of WT and APP/PS1 mice at 30 and 60 days after TBI. (**A**–**C**) Cortex, 30 days after TBI: *Il1b*, *Tnf*, and *Gsk3b*, respectively. (**D**–**F**) Hippocampus, 30 days after TBI: same gene panel. (**G**–**I**) Cortex, 60 days after TBI: same gene order. (**J**–**L**) Hippocampus, 60 days after TBI: same gene panel. WT, white bars; APP/PS1, grey bars. * *p* < 0.05, ** *p* < 0.01, *** *p* < 0.001 (two-way ANOVA).

While synuclein expression (*Snca*, *Sncb*, *Sncg*) remained relatively stable under most conditions and *Gfap* showed predominantly genotype-dependent effects, we further examined markers of active neuroinflammatory processes. Figure 3 presents the relative normalised expression of *Il1b*, *Tnf*, and *Gsk3b* in the cortex and hippocampus across the same experimental groups.

In the cortex, 30 days after TBI, a significant genotype effect on *Il1b* expression was observed (F = 14.80, *p* = 0.0012; two-way ANOVA; Figure 3A). APP/PS1 mice exhibited increased Il1b expression compared with WT animals under both control and TBI conditions. In contrast, *Tnf* expression showed no significant genotype effect (F = 0.001118, *p* = 0.9737; Figure 3B), although *Tnf* levels were numerically higher in APP/PS1 mice than in WT animals in both experimental conditions. For *Gsk3b* expression, an opposite pattern was observed (Figure 3C), with a significant decrease in APP/PS1 control mice compared with WT controls. A similar trend was observed following TBI; however, this difference did not reach statistical significance.

In the hippocampus, 30 days after TBI, a significant effect of genotype on *Il1b* expression was observed (F = 20.50, *p* = 0.0003; two-way ANOVA; Figure 3D). APP/PS1 mice exhibited elevated *Il1b* expression compared with WT animals under both control and TBI conditions. For *Tnf* expression a similar trend toward increased levels in APP/PS1 mice was observed although it did not reach statistical significance (Figure 3E). For *Gsk3b* expression a near-significant effect of TBI was detected (F = 4.095, *p* = 0.0601; Figure 3F) with WT mice subjected to TBI showing increased *Gsk3b* expression compared with WT controls.

In the cortex, 60 days after TBI, no significant effect of TBI on *Il1b* expression was detected (F = 1.251, *p* = 0.2740; two-way ANOVA; Figure 3G). *Il1b* expression was reduced in WT mice subjected to TBI compared with WT controls. A non-significant effect of genotype was also observed (F = 1.552, *p* = 0.2244). However, APP/PS1-TBI mice exhibited higher *Il1b* expression than WT-TBI animals, consistent with the pattern observed at earlier time points. No significant differences between groups were detected for *Tnf* expression (Figure 3H). For *Gsk3b* expression a significant effect of TBI was identified (F = 4.607, *p* = 0.0410; Figure 3I) with WT-TBI mice showing increased expression compared with WT controls. In addition, a significant effect of genotype was observed in control groups (F = 4.529, *p* = 0.0426) with APP/PS1 control mice exhibiting higher *Gsk3b* expression than WT controls.

In the hippocampus, 60 days after TBI, no significant differences between groups were observed for *Il1b*, *Tnf* and *Gsk3b* expression levels (Figure 3J–L).

### 3.2. Behavioural Testing

The “Open Field” test

An “Open field” test was performed to assess locomotor activity and exploratory behaviour as well as anxiety levels in mice. The analysis was performed at two age points: 30 days after TBI and 60 days after TBI. The parameters of locomotion (the average speed of movement and the total distance travelled) and the parameters associated with anxiety (the number of visits to the central zone and the time spent in the centre) were evaluated. Statistical analysis revealed no significant differences between the groups in any of the measured parameters at the first follow-up period one month after the injury.

At two months post-injury, progression of amyloid pathology would be expected in transgenic groups. However, analysis of Open Field test data revealed no significant differences between groups. These findings suggest that, under the present experimental conditions, neither genotype, TBI nor their interaction significantly affected locomotor activity or anxiety-related behaviour in mice. The absence of significant differences was observed at both one and two months post-injury.

Overall, these findings suggest that motor activity and anxiety-related behaviour remained largely preserved at these time points.

The “Novel Object Recognition” test

The “Novel Object Recognition” test is designed to assess declarative memory in rodents based on an innate preference to explore new objects. The study evaluated two main indicators: the preference index and the discrimination index.

**Figure 4:**
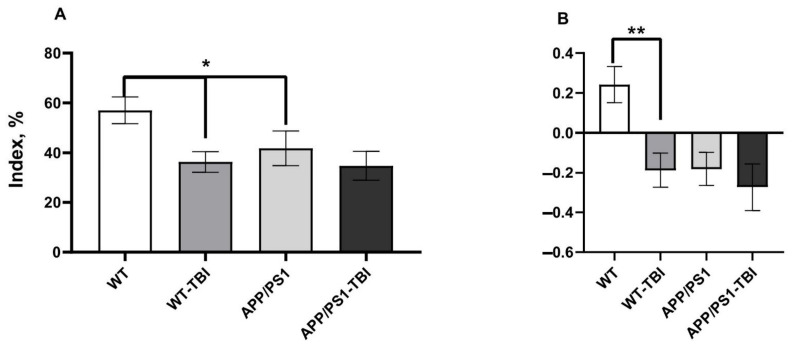
Novel object recognition performance 30 days after TBI (7 months of age). Novel object preference index (**A**), discrimination index (**B**). Abbreviations: APP/PS1 and WT, control groups; APP/PS1-TBI and WT-TBI, TBI groups. Values are presented as mean ± SEM (*n* = 9 per group; 4 males and 5 females). *—*p* < 0.05, **—*p* < 0.01 (one-way ANOVA).

In the early post-traumatic period, the preference index was significantly higher in intact mice compared with the injured groups (WT-TBI and APP/PS1-TBI) (F = 3.33, *p* = 0.0194, one-way ANOVA; Figure 4A). This suggests that as early as one-month post-TBI healthy animals show a reduced preference for a novel object while in transgenic mice injury is associated with an additional impairment. At this time point, APP/PS1 mice did not differ from WT animals in this measure. The discrimination index was also significantly affected with TBI in WT mice leading to a reduction in performance (F = 5.672, *p* = 0.0051; one-way ANOVA; Figure 4B).

**Figure 5:**
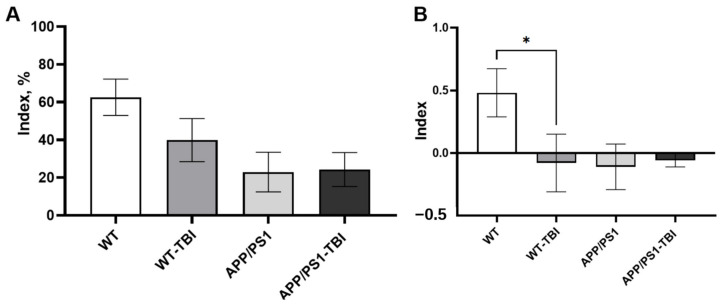
Novel object recognition performance 60 days after TBI (8 months of age). Novel object preference index (**A**), discrimination index (**B**). Abbreviations: APP/PS1 and WT, control groups; APP/PS1-TBI and WT-TBI, TBI groups. Values are presented as mean ± SEM (*n* = 9 per group; 4 males and 5 females). *—*p* < 0.05 (one-way ANOVA).

Thus, as early as 30 days after TBI, APP/PS1 mice exhibited cognitive deficits reflected in a reduced discrimination index. TBI further impaired performance in WT mice; whereas, no statistically significant effect was observed in APP/PS1 mice (F = 5.672, *p* > 0.05; one-way ANOVA; Figure 4B). A different pattern emerged in the delayed post-traumatic period with differences in the preference index between injured and control groups no longer reaching statistical significance: WT vs. WT-TBI (F = 3.392, *p* = 0.1439; Figure 4A) and APP/PS1 vs. APP/PS1-TBI (F = 3.392, *p* = 0.9264; Figure 5A). At 60 days after TBI, the difference in the discrimination index between WT and WT-TBI mice remained statistically significant (F = 2.464, *p* = 0.0318; Figure 5B); whereas, no significant difference was observed between APP/PS1 and APP/PS1-TBI mice (F = 2.464, *p* = 0.8493; Figure 5B). Thus, trauma-associated impairment persisted in WT mice, while in APP/PS1 mice the effect of TBI was less distinguishable against the background of the transgenic phenotype.

The Y-maze test

The “Y-maze” test assessed both general locomotor activity and short-term memory.

**Figure 6:**
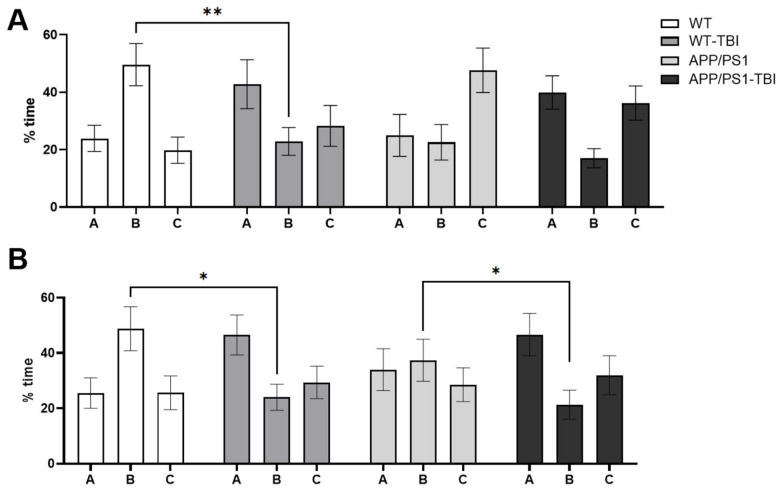
Y-maze test performance 30 days after TBI (7 months of age). (**A**) Percentage of “new” arm selection compared to the “old” one in the first 2 min of the test. (**B**) Percentage of the “new” arm selection compared to the “old” one in 5 min of the test. Abbreviations: APP/PS1 and WT, control groups; APP/PS1-TBI and WT-TBI, TBI groups; A—starting arm; B—novel arm; C—familiar arm. Values are presented as mean ± SEM (*n* = 9 per group; 4 males and 5 females). * *p* < 0.05, ** *p* < 0.01 (percentage time spent in the new arm; one-way ANOVA).

At both time points no statistically significant differences were observed between groups in either total distance travelled or mean velocity (*p* > 0.05). These findings indicate that the behavioural differences observed in the test are unlikely to be attributable to impairments in motor function and may therefore be interpreted as reflecting cognitive effects. A pronounced impairment in spatial memory was observed in TBI mice compared with WT controls during the first 2 min of the test (WT vs. WT-TBI: F(6, 192) = 5.719, *p* = 0.0084; one-way ANOVA; Figure 6A). In contrast, no significant differences were detected between APP/PS1 and APP/PS1-TBI groups (F(6, 192) = 5.719, *p* = 0.7976); however, a non-significant trend towards impaired short-term memory was noted in the injured transgenic mice.

When the test duration was extended to 5 min the TBI groups retained a reduced proportion of visits to the novel arm (F(6, 183) = 3.054; WT-TBI: *p* = 0.0262; APP/PS1-TBI: *p* = 0.2049; one-way ANOVA; Figure 6B) suggesting a more persistent TBI-induced impairment in memory function. Thus, the effects of genotype and trauma on short-term memory were comparable and did not appear to be additive.

**Figure 7:**
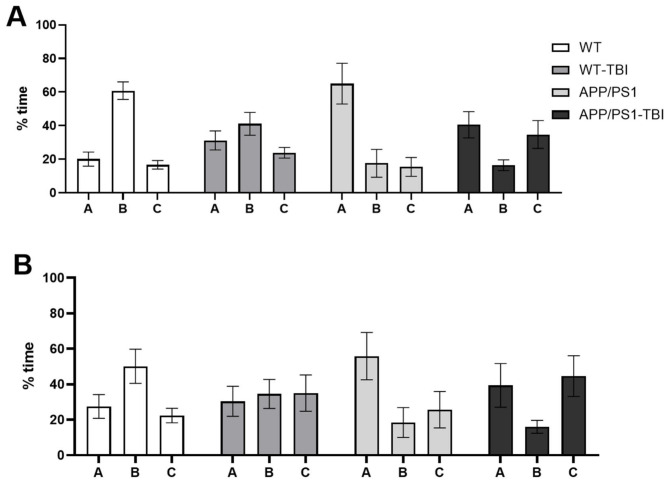
Y-maze test performance 60 days after TBI (8 months of age). (**A**) Percentage of “new” arm selection compared to the “old” one in the first 2 min of the test. (**B**) Percentage of the “new” arm selection compared to the “old” one in 5 min of the test. Abbreviations: APP/PS1 and WT, control groups; APP/PS1-TBI and WT-TBI, TBI groups; A—starting arm; B—novel arm; C—familiar arm. Values are presented as mean ± SEM (*n* = 9 per group; 4 males and 5 females). *p* > 0.05 (one-way ANOVA).

At the second time point of the experiment TBI in WT mice resulted in a disruption of directed arm choice (A–B, B–C, C–B; *p* > 0.05), indicating that arm selection occurred at random without clear novelty avoidance. In APP/PS1 mice subjected to TBI the previously observed pathological preference for the starting arm was no longer evident (F(6, 96) = 10.13, *p* = 0.4814; one-way ANOVA; Figure 7A). However, the low preference for the novel arm persisted and did not differ significantly from APP/PS1 controls (F(6, 96) = 10.13, *p* = 0.9982; one-way ANOVA; Figure 7A). The pattern observed after 5 min did not differ substantially from that seen at 2 min (Figure 7B); mice subjected to TBI showed no clear arm preference suggesting impairment of episodic-like memory.

The WT group showed stable spatial memory at both time points, with no evidence of age-related decline. TBI-induced impairment in spatial memory was still present two months after injury; although, it was reduced compared with the earlier post-injury stage. In APP/PS1 mice, cognitive performance declined with age. At 30 days after TBI, these animals still showed some preference for exploring the novel arm at the 5 min interval; however, by 60 days after TBI, they mainly remained in the starting arm even during longer testing, indicating a stronger bias towards the familiar location rather than novelty exploration. APP/PS1-TBI mice showed a tendency towards further short-term memory impairment, but this was not significantly different from APP/PS1 controls.

The “Barnes Maze” Test

The Barnes Maze test assesses spatial learning and memory based on the ability to locate an escape hole using distal spatial cues. During the first four days of training distance travelled, movement speed (as an indicator of motor activity) and latency to find the target hole were recorded.

Across all training days at both time points no statistically significant differences were observed between groups in either speed or distance travelled (*p* > 0.05; mixed-effects model (REML)). The absence of group differences in locomotor parameters indicates that changes in other behavioural measures can be interpreted as reflecting cognitive rather than motor effects.

The WT group exhibited typical spatial learning. Latency decreased significantly from day 1 to days 2, 3, and 4 (all *p* < 0.0001). The absence of significant differences between days 2, 3, and 4 (*p* > 0.05) indicates a performance plateau consistent with learning consolidation.

The WT-TBI group showed a similar pattern. Latency was significantly reduced from day 1 to day 2 (*p* < 0.0001), day 3 (*p* = 0.0004), and day 4 (*p* = 0.0002). No significant differences were observed between days 2, 3, and 4 (*p* > 0.05) suggesting that learning was preserved despite injury.

The APP/PS1 group exhibited markedly impaired learning performance. A significant reduction in latency was observed only when comparing day 1 with day 3 (*p* = 0.0009) and day 1 with day 4 (*p* < 0.0001). However, no significant differences were detected between days 1 and 2 (*p* = 0.383) or between days 2 and 3 (*p* = 0.2743) indicating an absence of early learning progression. Although some improvement was evident by day 4 it occurred with a pronounced delay suggesting impaired spatial learning and reduced learning capacity in APP/PS1 mice.

The APP/PS1-TBI group showed a complete absence of learning across the training period. No significant differences were observed between any training days (day 1 vs. day 2: *p* = 0.7984; day 1 vs. day 3: *p* = 0.1344; day 1 vs. day 4: *p* = 0.2323; all *p* > 0.05) and latency remained consistently elevated throughout the task.

**Figure 8:**
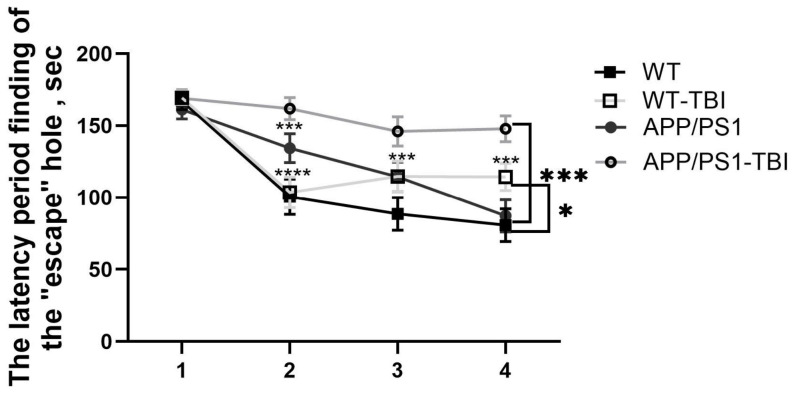
Performance across training days in the “Barnes Maze” (30 days after TBI, 7 months of age). The latency period finding of the “escape” hole. The *X*-axis represents the days of training in the Barnes test. Abbreviations: APP/PS1 and WT, control groups; APP/PS1-TBI and WT-TBI, TBI groups. Values are presented as mean ± SEM (*n* = 9 per group; 4 males and 5 females). * *p* < 0.05, *** *p* < 0.001, **** *p* < 0.0001 (one-way ANOVA post hoc comparisons).

Comparison between groups showed no significant differences on the first day (F(9, 420) = 4.054, *p* > 0.05; two-way ANOVA; Figure 8) indicating comparable baseline performance across all groups. In contrast, differences between control and TBI groups emerged on the final day of training. (F (9. 420) = 4.054, WT-TBI: *p* = 0.0491, APP/PS1-TBI: *p* = 0.0002, Two-way ANOVA, Figure 8).

**Figure 9:**
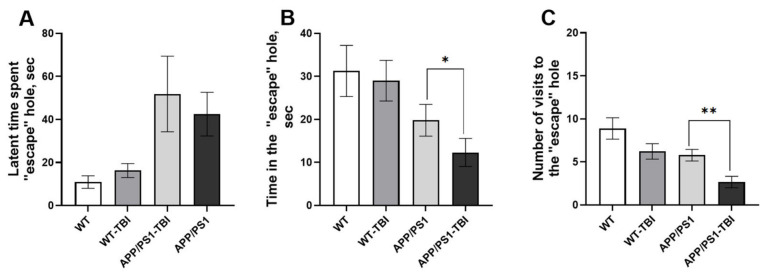
Performance on the 5th test day of the Barnes Maze (30 days after TBI, 7 months of age). The latent time spent in the “escape” hole (**A**), the time spent in the “escape” hole (**B**), the number of visits to the “escape” hole (**C**). Abbreviations: APP/PS1 and WT, control groups; APP/PS1-TBI and WT-TBI, TBI groups. Values are presented as mean ± SEM (*n* = 9 per group; 4 males and 5 females). * *p* < 0.05, ** *p* < 0.01 (one-way ANOVA).

The APP/PS1-TBI group exhibited more pronounced impairments compared with APP/PS1 controls as reflected in both the number of visits to the escape hole (F = 8.003, *p* = 0.0023; one-way ANOVA; Figure 9C) and the total time spent in the escape zone (F = 3.715, *p* = 0.0453; one-way ANOVA; Figure 9B). In contrast, the WT-TBI group did not differ significantly from WT controls across any measured parameters (F = 8.003, *p* = 0.1890; F = 3.715, *p* = 0.9253; F = 3.661, *p* = 0.9201; one-way ANOVA; Figure 9) indicating preserved spatial learning in healthy animals following traumatic brain injury.

Thus, by the fifth day of training the most pronounced cognitive impairment was observed in APP/PS1-TBI mice; whereas, TBI alone did not induce detectable deficits in WT animals. In contrast, the combination of amyloid pathology and injury resulted in a moderate increase in latency indicating an additive but not synergistic effect on spatial learning performance.

At the second time point the learning profile showed a pattern broadly similar to that observed in both APP/PS1 and WT groups. No significant differences were detected between APP/PS1-TBI and APP/PS1 mice across all training days (F(9, 408) = 2.697, *p* > 0.05; two-way repeated-measures ANOVA). The largest difference was observed on day 2 (Δ = 20.18, *p* = 0.1457); although, this did not reach statistical significance.

**Figure 10:**
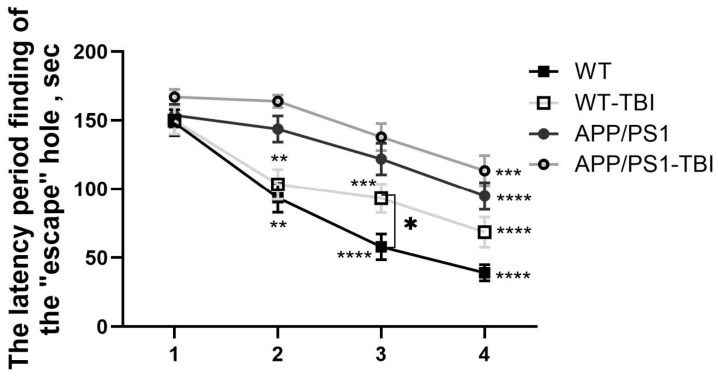
Performance across training days in the “Barnes Maze” (60 days after TBI, 8 months of age). The latency period finding of the “escape” hole. The *X*-axis represents the days of training in the Barnes test. Abbreviations: APP/PS1 and WT, control groups; APP/PS1-TBI and WT-TBI, TBI groups. Values are presented as mean ± SEM (*n* = 9 per group; 4 males and 5 females). * *p* < 0.05, ** *p* < 0.01, *** *p* < 0.001, **** *p* < 0.0001. (one-way ANOVA post hoc comparisons).

In WT animals no differences between WT-TBI and WT groups were observed during the first two days of training (F(9, 408) = 2.697, *p* > 0.05). However, a significant increase in latency was detected in the injured group on day 3 (Δ = 35.33 s, *p* = 0.0354) with a non-significant trend persisting on day 4 (*p* = 0.0578; Figure 10). These findings indicate that TBI in healthy mice induces a delayed impairment in spatial learning becoming evident during the acquisition phase and suggesting a slowing of skill formation rather than an immediate learning deficit.

The WT group demonstrated classical spatial learning. A significant decrease in latency was revealed when comparing day 1 with day 2 (*p* = 0.0018), 3 (*p* < 0.0001) and 4 (*p* < 0.0001) as well as day 2 with day 3 (*p* = 0.0234) and 2 with 4 (*p* = 0.0011). The absence of differences between days 3 and 4 (*p* = 0.3917) indicates skill stabilisation.

The WT-TBI group demonstrated effective learning which evidenced by a significant reduction in latency when comparing day 1 with days 2 (*p* = 0.0037), 3 (*p* = 0.0008) and 4 (*p* < 0.0001), as well as between days 2 and 4 (*p* = 0.0373). The absence of significant differences between days 2 and 3 and between days 3 and 4 (both *p* > 0.05) suggests stable performance and successful memory formation.

In contrast, the APP/PS1 group showed a marked impairment in learning ability. Although a significant reduction in latency was observed by the end of training when comparing day 1 with day 4 (*p* < 0.0001) and day 2 with day 4 (*p* = 0.0007) no significant differences were detected between days 1 and 2 (*p* = 0.7753), days 2 and 3 (*p* = 0.2674) and days 3 and 4 (*p* = 0.0699). This indicates a lack of meaningful improvement during the first three days of training. An improvement is only evident by day 4; however, the absence of progressive change across earlier sessions suggests a substantial impairment in the formation of spatial memory.

The APP/PS1-TBI group showed a continuing marked impairment in learning ability despite a slight reduction in the deficit relative to the first time point. Although a significant decrease in latency was observed when comparing day 1 with day 4 (*p* = 0.0006) and day 2 with day 4 (*p* = 0.0001). No significant differences were detected between day 1 and day 2 (*p* = 0.9521), day 1 and day 3 (*p* = 0.0559), day 2 and day 3 (*p* = 0.0592). This indicates a lack of statistically significant progress during the first three days of training. Any improvement is only evident by day 4; however, the very slow rate of change in performance suggests a substantial impairment in spatial learning and a persistently reduced learning capacity.

**Figure 11:**
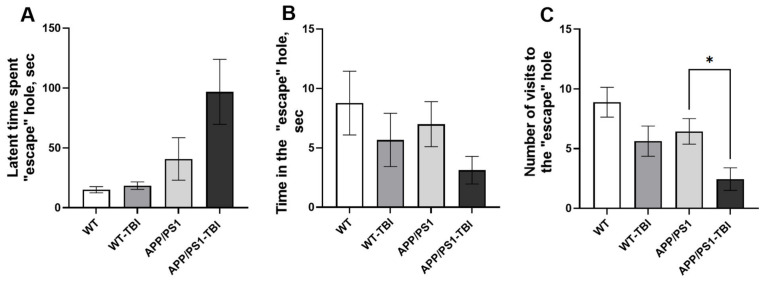
Performance on the 5th test day of the Barnes Maze (60 days after TBI, 8 months of age). The latent time spent in the “escape” hole (**A**), the time spent in the “escape” hole (**B**), the number of visits to the “escape” hole (**C**). Abbreviations: APP/PS1 and WT, control groups; APP/PS1-TBI and WT-TBI, TBI groups. Values are presented as mean ± SEM (*n* = 9 per group; 4 males and 5 females). *—*p* < 0.05 (one-way ANOVA).

At the second time point the severity of long-term memory impairment in the APP/PS1-TBI group was reduced compared with the APP/PS1 group. Only one parameter remained significantly different, namely the number of visits to the escape hole (F = 5.654, *p* = 0.0333, one-way ANOVA; Figure 11C). The WT-TBI group did not differ from the WT group in any of the assessed parameters.

At this stage, APP/PS1 mice showed a progression of cognitive deficits and were significantly impaired compared with WT mice from day 2 onwards across all stages of learning. Traumatic brain injury did not lead to additional long-term impairment in the transgenic animals. This may reflect a ceiling effect whereby performance is already substantially compromised or alternatively suggests that the effects of trauma and genetic pathology are not additive at this age.

### 3.3. Histological Examination of the Cerebral Cortex and Hippocampus

**Figure 12:**
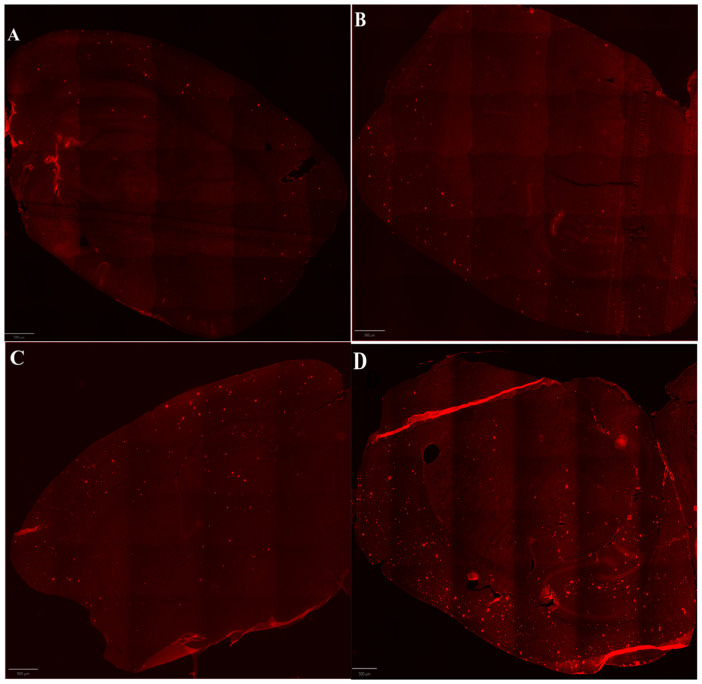
Histological analysis of Aβ inclusions on brain slices of APPswe/PS1dE9/Blg transgenic animals and wild-type control animals 30 and 60 days after TBI; Congo Red coloration. The measurement scale is 500 microns. APP/PS1-TBI (30 days after TBI) (**A**), APP/PS1 (**B**) (7 month of age), APP/PS1-TBI (60 days after TBI) (**C**), APP/PS1 (8 month of age) (**D**).

To assess Aβ amyloid pathology in the cerebral cortex and hippocampus of experimental mice Congo red staining was used on brain sections. Amyloid plaques were visualised as red deposits. The data illustrate the combined influence of genetic factors (APP/PS1 expression level) and external injury (TBI) on amyloid plaque formation across different brain regions (Figure 12).

**Figure 13:**
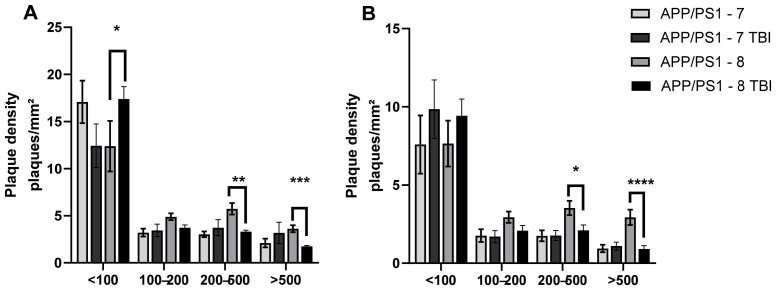
Size distribution of amyloid plaques in the cerebral cortex and hippocampus at two experimental time points. (**A**) Plaque size distribution in the cerebral cortex; (**B**) plaque size distribution in the hippocampus. The plaques are arranged in the following gradation: <100 µm^2^, 100–200 µm^2^, 200–500 µm^2^, and >500 µm^2^. Plaque size categories are expressed in µm^2^. Plaque density was calculated as the number of plaques per analysed tissue area and expressed as plaques/mm^2^. Values are presented as mean ± SEM (*n* = 9, per group; 4 males and 5 females). * *p* < 0.05, ** *p* < 0.01, *** *p* < 0.001, **** *p* < 0.0001. (Sidak’s multiple comparisons test following two-way ANOVA).

Exactly 30 days after TBI, no statistically significant differences were observed between APP/PS1 mice with TBI and APP/PS1 mice without TBI in either the cerebral cortex or hippocampus (Figure 13).

At 60 days after TBI, in the cerebral cortex, the APP/PS1 group (positive control) exhibited a significantly higher number of small amyloid deposits (<100 µm^2^) compared with APP/PS1-TBI mice (*p* = 0.0155; two-way ANOVA). A non-significant trend towards a reduced number of medium-sized inclusions (100–200 µm^2^) was also observed in the TBI group (*p* = 0.087). In addition, the APP/PS1-TBI group showed a significant reduction in the number of large amyloid deposits (>500 µm^2^) compared with APP/PS1 controls (*p* = 0.0001; two-way ANOVA).

In the hippocampus, 60 days after TBI, the APP/PS1 (positive control) group displayed significantly fewer deposits in the 200–500 µm^2^ range compared with APP/PS1-TBI mice (*p* = 0.00209, two-way ANOVA), as well as a marked reduction in large deposits (>500 µm^2^; *p* < 0.0001, two-way ANOVA).

## 4. Discussion

The choice of the APPswe/PS1dE9/Blg transgenic line for the present study was based on several considerations. First, this model is widely regarded as one of the most relevant experimental models of AD as it recapitulates the age-dependent accumulation of β-amyloid plaques within the cortex and hippocampus [44]. This feature enables the investigation of the effects of TBI at different stages of disease progression. Furthermore, APPswe/PS1dE9/Blg mice develop marked deficits in learning and memory, thereby providing an opportunity to assess the interaction between TBI-induced neuropathological changes and cognitive dysfunction. The presence of robust and reproducible amyloid pathology together with well-characterised behavioural impairments makes this model particularly suitable for examining how TBI influences synuclein-associated transcription, amyloid plaque morphology and cognitive performance in the context of AD-related neurodegeneration [36].

A key advantage of the APPswe/PS1dE9/Blg line over other transgenic models, particularly the 5xFAD line, is the absence of pronounced α-synuclein overexpression. In 5xFAD mice, increased α-synuclein expression has been reported to accompany amyloid plaque formation, potentially introducing an additional pathological component that may confound the interpretation of findings attributable specifically to amyloid pathology. The use of APPswe/PS1dE9/Blg mice therefore permits a more focused evaluation of the effects of TBI on Aβ aggregation and plaque morphology while minimising the confounding influence of concomitant synucleinopathy [46].

The absence of marked acceleration of β-amyloid pathology following TBI observed in the present study is consistent with previous experimental findings indicating that brain injury does not necessarily result in a sustained increase in amyloid burden in transgenic models of AD. In a recent study using APPswe/PS1dE9/Blg mice repeated mild TBI induced persistent neuronal damage as evidenced by elevated neurofilament light chain (NF-L) levels, but did not produce a significant increase in amyloid deposition one month after injury. These findings are in agreement with our results obtained over a comparable post-injury interval [23]. This concordance suggests that TBI may not be sufficient to exacerbate β-amyloid accumulation during the early stages following trauma despite inducing neuronal injury.

However, this pattern is not consistent across studies as the effects of TBI on Aβ pathology appear to depend on the age of the animals, the stage of amyloidogenesis and the severity of the injury. In the tg-ArcSwe model TBI induced before the onset of amyloid pathology accelerated Aβ accumulation at later but not earlier time points, an effect associated with delayed glial activation [47]. Similarly, in APPswe/PS1dE9/Blg mice with established transgene-driven pathology TBI increased hippocampal amyloid deposition and was accompanied by memory deficits and neuronal loss in the CA3 region, effects attributed primarily to a shift in microglia towards the pro-inflammatory M1 phenotype [48]. Our findings observed in the presence of pre-existing amyloid pathology support the concept that the effects of TBI on amyloidogenesis are stage- and context-dependent. Moreover, they suggest that the behavioural and morphological consequences of TBI may not necessarily parallel changes in overall β-amyloid burden.

The findings related to the synuclein family provide an additional perspective on the interaction between TBI and Alzheimer-like pathology. Although *Snca*, *Sncb*, and *Sncg* expression did not exhibit consistent injury-induced changes across brain regions or time points, the increased cortical expression of *Sncg* observed in APPswe/PS1dE9/Blg mice suggests that amyloid pathology may be associated with a distinct synuclein-related transcriptional profile. In contrast, the absence of similar changes in the hippocampus indicates that these responses are region-specific rather than widespread and are likely influenced by local pathological conditions and differential regional vulnerability. From a mechanistic perspective, *Sncg* encodes γ-synuclein, a protein implicated in the KEGG Neurodegeneration—multiple diseases pathway through processes shared with α-synuclein, including protein misfolding and aggregation, impairment of proteasomal function, and mitochondrial stress [49,50]. In addition, γ-synuclein has been reported to activate the MAPK/Elk-1 signalling pathway and to interact with the MKK3/6–p38 MAPK axis, both of which regulate stress-responsive pro-inflammatory and pro-apoptotic transcriptional pathways [51,52]. These observations suggest that the increased cortical expression of *Sncg* detected in APPswe/PS1dE9/Blg mice may reflect the engagement of molecular pathways associated with neurodegenerative and cellular stress responses.

Moreover, in the context of amyloid pathology members of the synuclein family including γ-synuclein have been implicated in the aggregation and intercellular propagation of synuclein species via vesicle- and exosome-mediated prion-like mechanisms which may interact with β-amyloid-driven pathological processes [53,54]. Collectively, these observations provide a plausible mechanistic framework for the region-specific and genotype-dependent increase in *Sncg* expression observed in the cortex of APPswe/PS1dE9/Blg mice. However, these observations are derived from transcriptomic analyses and therefore should be interpreted as associative rather than causal. Importantly, our data do not support a causal role for synucleins in amyloid remodelling or cognitive impairment. Rather, alterations in synuclein gene expression should be regarded as associated molecular changes that warrant further investigation at the protein level and should be examined in relation to plaque morphology, synaptic integrity, and behavioural outcomes.

Behavioural changes observed in both APPswe/PS1dE9/Blg and wild-type mice one month after TBI indicate the presence of trauma-induced dysfunction that occurs independently of any substantial increase in amyloid pathology. Comparable findings have been reported in models of mild and repetitive TBI in which cognitive impairment and disrupted hippocampal function were evident despite no significant increase in either the number or the overall area of Aβ plaques [23,25,55].

By the two-month follow-up the behavioural profile of APPswe/PS1dE9/Blg mice in the present study was determined primarily by the progression of the underlying transgenic pathology; whereas, the contribution of TBI appeared less pronounced. This is in line with evidence suggesting that brain injury mainly alters the temporal onset of cognitive impairment and synaptic dysfunction in AD models without necessarily modifying the eventual level of amyloid burden in an already established pathological cascade [24,48]. Overall, these findings underscore that behavioural outcomes following TBI in AD models reflect a complex interaction between primary injury, synaptic and vascular dysfunction, neuroinflammatory processes and pre-existing neurodegeneration rather than being directly proportional to β-amyloid load.

A notable finding of the present study is the alteration in the morphological profile of amyloid pathology following TBI characterised by a reduction in the average plaque size accompanied by an increase in plaque number. This pattern suggests a remodelling of the cerebral amyloid pool rather than a simple linear accumulation of Aβ. Similar observations have been reported in both clinical and neuropathological studies of TBI where injury was associated with the rapid emergence of numerous small Aβ deposits morphologically resembling the early stages of amyloid plaque formation observed in AD. These findings support the notion that TBI may influence not only the extent of amyloid deposition but also the dynamics and organisation of amyloid aggregation [56,57].

A growing body of evidence suggests that traumatic injury can influence not only the overall burden of amyloid pathology but also the structural organisation and turnover of existing plaques. A number of studies have demonstrated that when injury occurs in the presence of established amyloid pathology plaque density may be reduced within the affected region. Mechanistically this phenomenon has been linked to astrocytic activation, enhanced proteolytic degradation of Aβ, and the induction of amyloid-clearing neuropeptidases. For example, in a spinal cord injury model using 5xFAD mice with pre-existing amyloid pathology a marked reduction in plaque burden within the injured area was observed as early as seven days post-injury [58]. This effect was preceded by early astrocytic activation and increased expression of amyloid-degrading enzymes followed by microglia-mediated phagocytic clearance of amyloid deposits [26,58]. Collectively, these findings indicate that tissue injury may trigger active remodelling of the plaque population through mechanisms that promote both amyloid clearance and redistribution.

Within this context our observation of a predominance of smaller plaques together with only a moderate increase in total Aβ burden may reflect trauma-induced restructuring of the plaque population. Rather than representing a simple acceleration of amyloid accumulation these changes are consistent with a dynamic process involving concurrent plaque formation, fragmentation and clearance, ultimately reshaping the morphological landscape of amyloid pathology following injury.

The pronounced glial response observed in the present study, characterised by reactive astrocytic hypertrophy and increased *Gfap* expression, further supports the concept that neuroinflammation plays a pivotal role in shaping Aβ pathology following TBI. As a hallmark of reactive astrogliosis, GFAP expression is tightly regulated by cytokine-dependent signalling networks, including cytokine–cytokine receptor interaction pathways and JAK–STAT3 signalling, both of which are recognised drivers of astrocyte reactivity in neurodegenerative disorders [59]. A substantial body of experimental and clinical evidence indicates that TBI triggers persistent activation of astrocytes and microglia, resulting in sustained production of pro-inflammatory cytokines such as IL-1β, IL-6, and TNF-α [60,61]. Beyond their effects on neuronal plasticity and functional recovery, these mediators have been shown to influence APP processing and Aβ metabolism [62,63]. It is therefore plausible that the enhanced astrogliotic response observed in our study contributes directly to the trauma-induced remodelling of amyloid pathology through inflammatory mechanisms that regulate both Aβ production and clearance.

Previous experimental studies have demonstrated that activation of hippocampal inflammatory pathways including increased cyclooxygenase-2 (COX-2) expression is associated with behavioural vulnerability following brain injury [64]. Furthermore, in both cellular and animal models, the combination of mechanical trauma and pre-existing Aβ pathology has been shown to induce pronounced astrocytic activation elevated GFAP expression, impaired autophagic function and increased neuronal susceptibility to injury [28]. These findings support the notion that neuroinflammatory processes may play a central role in mediating the interaction between traumatic injury and amyloid pathology.

In the context of the present study it is plausible that trauma-induced inflammation and the accompanying GFAP-associated astrocytic response contribute to the remodelling of the existing amyloid plaque population. Such mechanisms may promote the formation of numerous small Aβ deposits while maintaining or only moderately increasing the overall amyloid burden. Indirect support for this interpretation comes from studies demonstrating enhanced amyloidogenic processing of amyloid precursor protein (APP) together with the accumulation of small plaques in the acute phase following TBI, in the absence of a proportional increase in mature plaque burden. Collectively, these observations suggest that neuroinflammation may influence not only the quantity of Aβ deposition but also the morphological evolution and spatial organisation of amyloid pathology after injury.

### Limitations of the Study

This study has several limitations that should be considered when interpreting the findings. First, the observation period was restricted to two relatively early post-injury time points precluding assessment of the long-term effects of TBI on amyloid pathology, synuclein expression and cognitive decline during later stages of disease progression. Second, only a single TBI paradigm was employed; consequently, the findings cannot be readily generalised to other injury models such as repeated mild TBI or diffuse brain injury which may more closely reflect certain clinical scenarios. Third, inflammatory and synuclein-related alterations were assessed exclusively at the mRNA level. Validation at the protein level using techniques such as Western blotting, ELISA and immunohistochemistry will be necessary to establish the functional significance of the observed transcriptional changes. Fourth, amyloid pathology was evaluated using Congo red staining which primarily detects fibrillar amyloid deposits and does not capture soluble Aβ species or diffuse plaque pathology. Finally, although both male and female mice were included the study was not specifically powered to detect sex-dependent effects.

Despite these limitations the study provides novel evidence that TBI influences the morphological organisation of amyloid pathology and is associated with neuroinflammatory responses and altered synuclein expression in APPswe/PS1dE9/Blg mice. Future studies incorporating longer follow-up periods, additional injury paradigms, protein-level analyses and comprehensive assessment of soluble and insoluble Aβ species will be important for clarifying the mechanisms linking TBI to the progression of Alzheimer’s disease-related pathology.

## 5. Conclusions

In the present study TBI did not result in a uniform or linear increase in β-amyloid deposition in APPswe/PS1dE9/Blg mice with established amyloid pathology. Rather the findings suggest that traumatic injury alters the morphological characteristics of amyloid deposits particularly plaque-size distribution indicating a remodelling of the existing amyloid pool rather than a simple increase in overall Aβ burden. Cognitive outcomes appeared to depend on the interplay between injury-related processes and the progression of the underlying APPswe/PS1dE9/Blg phenotype. At the early post-injury stage TBI was associated with measurable impairments in memory-related behavioural tasks; whereas, at the later time point behavioural performance appeared to be driven predominantly by the progression of the transgenic disease process.

Gene-expression analyses further revealed region- and genotype-specific alterations in synuclein-family transcripts and neuroinflammation-related markers with the most pronounced changes observed in the cerebral cortex. Together with the observed astrocytic response these findings support the hypothesis that neuroinflammatory mechanisms may contribute to trauma-induced remodelling of amyloid pathology and influence disease progression. Overall, our results suggest that the effects of TBI on amyloid pathology in APPswe/PS1dE9/Blg mice are context-dependent and may involve changes in plaque morphology and local tissue responses rather than a uniform increase in total amyloid burden. These effects appear to depend on the stage of pre-existing pathology and the regional response to injury.

Further studies incorporating sham-operated controls, extended follow-up periods, protein-level validation and detailed histopathological assessment of glial and amyloid-related markers will be required to elucidate the exact cellular and molecular mechanisms underlying these interactions and to determine their relevance to the progression of Alzheimer’s disease following traumatic brain injury.

## Figures and Tables

**Table 1:** Sequences of primers used for RT-PCR.

Gene Name	Gene	Direction	Primer Sequence
*Gapdh*	glyceraldehyde 3-phosphate dehydrogenase	F R	ATG ACC ACA GTC CAT GCC ATCGAG CTT CCC GTT CAG CTC TG
*Snca*	alpha-synuclein	F R	CTG CCC TTG CCT CTT TCA TTG TGA ACA CAT CCA TGG CTA AAG A
*Sncb*	beta-synuclein	F R	CAA GGA AGG CGT CCT CTA TGT ATG CCT GCT CCT TGG TTT TCT
*Sncg*	gamma-synuclein	F R	GGG GTT CCA AGT CCT CCT T CAA CAC AGT GGC CAA CAA GA
*Gfap*	glial fibrillary acidic protein	F R	GCC TCC TCA CCT ATG CAC TC CCC AGA GAT GCA AGT CCA AT
*Il1b*	Interleukin-1beta	F R	CCA CAT TGA CCA GAC AGG GG TCT TCA TGC CCA AAG CAG GT
*Tnf*	tumour necrosis factor alpha	F R	ATC CCA GGA CCT TAT CCC CC GTG TCT GGA AGG TAG CGG TC
*Gsk3b*	glycogen synthase kinase-3 beta	F R	GGC CTT CAC TGG ATC AAC AC GGG TGA CTC TCT TCA GAT TG

## Data Availability

All of the data that support the findings of this study are available in the main text.

## Abbreviations

The following abbreviations are used in this manuscript:

TBI	Traumatic brain injury
AD	Alzheimer’s disease
NDD	Neurodegenerative diseases
APP/PS1	APPswe/PS1dE9/Blg
